# Snakebite Envenoming a Challenging Diagnosis for the Forensic Pathologist: A Systematic Review

**DOI:** 10.3390/toxins12110699

**Published:** 2020-11-03

**Authors:** Alessandro Feola, Gian Luca Marella, Anna Carfora, Bruno Della Pietra, Pierluca Zangani, Carlo Pietro Campobasso

**Affiliations:** 1 Department Experimental Medicine, University of Campania “Luigi Vanvitelli”, via Luciano Armanni 5, 80138 Naples, Italy; alessandro.feola@unicampania.it (A.F.); bruno.dellapietra@unicampania.it (B.D.P.); pierluca.zangani@unicampania.it (P.Z.); carlopietro.campobasso@unicampania.it (C.P.C.); 2 Department of Surgical Sciences, University of Rome “Tor Vergata”, via Montpellier 1, 00133 Rome, Italy; glmarella@gmail.com

**Keywords:** snakebite envenomation, autopsy, toxicology, neglected tropical disease

## Abstract

Snakebite envenoming (SBE) is a public health issue in developing countries. The estimated annual global incidence of snakebites is about 5.4 million snakebites per year, resulting from 1.8 to 2.7 million cases of SBE and from 81,000 to 138,000 deaths with 400,000 survivors suffering permanent physical and psychological disabilities. There are more than 3000 species of snakes around the world: 600 are venomous and over 200 are considered to be medically important because of their clinical effects. The severity of SBE depends on several factors among which bite localization, snake’s size, condition of glands and teeth, bite angle and bite duration, the microflora of the snake’s mouth and victim’s skin, age of the victim, weight, health status, and victim’s activity after a bite. Snake venoms are mixtures of protein families, and each of these families contains many different toxins or toxin isoforms. Based on their effects, snake venoms can be classified as hemotoxic, neurotoxic, or cytotoxic and they can all act together involving multiple tissues and organs. When the bite is fatal, the mechanism of death is primarily related to the paralysis of respiratory muscles, which causes asphyxia and hypoxic-ischemic encephalopathy, but also anaphylactic shock, hemorrhagic shock, cardiomyopathy, acute tubular necrosis (ATN). The purpose of this literature review is to evaluate epidemiological and post-mortem examination findings in fatal SBEs in order to better understand the pathophysiological mechanisms, thus helping pathologists in defining the correct diagnosis.

## 1. Introduction

SBE is a potentially life-threatening disease resulting from the injection of a mixture of different toxins (venom) that particularly afflicts the poorest people living in developing countries with the worst quality of life [1]. In 2017, the World Health Organization (WHO) listed SBE as a priority neglected tropical disease (NTD). WHO reports 5.4 million snakebites per year, resulting from 1.8 to 2.7 million cases of SBE and from 81,000 to 138,000 deaths with 400,000 survivors suffering permanent physical and psychological disabilities [1,2]. The promptness of the therapy is a key element for a good outcome after SBE. However, given the socio-cultural and economic context of the territories, effective treatment is often delayed due to the lack of hospital infrastructure, the non-availability of a species-specific antivenom or delay in its injection caused by the not prompt identification of the offending snake, and the use of traditional healers or popular remedies [3,4]. Pathogenesis induced by snake venoms is complex and characterized by local and systemic alterations represented mostly by bleeding, dermo/myonecrosis, inflammation, and coagulation disorders. Mechanisms of toxicity remain poorly understood because the venoms are complex mixtures of components that induce different effects [5]. Snake venom can be detected post-mortem through many toxicological ways, such as bioassays, radioimmunoassays (RIA), fluorescence immunoassays, immunoelectrophoresis, ELISA (enzyme-linked immunosorbent assay) [6]. Cytotoxins, neurotoxins, and hemotoxins are commonly identified depending on the species of the offending snake.

There are more than 3000 species of snakes around the world: 600 are venomous and over 200 are considered to be medically important because of their clinical effects [7]. New species of snakes are still being discovered, and many species formerly recognized as being widespread have been found to comprise multiple separate species [8]. Although the majority of snake species do not have venom and kill their prey by squeezing them, snakes are classified by WHO guidelines [8,9] based on production, control, and regulation of snake antivenom immunoglobulins and according to risk assessment: in category 1 (highest medical importance) there are highly venomous snakes that are common or widespread and cause numerous snakebites, resulting in high levels of morbidity, disability or mortality; in category 2 (secondary medical importance) there are highly venomous snakes capable of causing morbidity, disability or death, for which exact epidemiological or clinical data may be lacking; and/or are less frequently implicated. The aim of this study is to evaluate retrospectively the characteristics of fatal SBE reported in the literature.

## 2. Results

### 2.1. Characteristics of Eligible Studies

**Figure 1:**
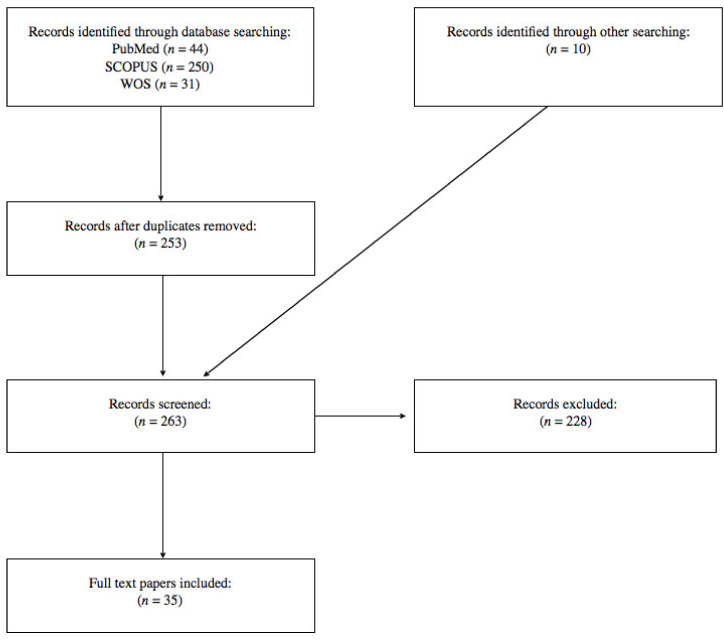
Flowchart depicting the choice of the studies.

The search of PubMed, Scopus, Web of Science, and Google Scholar databases provided a total of 263 articles. After adjusting for duplicates and reviewing titles and abstracts, 228 studies were discarded. Finally, only 35 papers reporting, in total, 56 cases of fatal SBE satisfied the inclusion criteria. A flowchart depicting the selection of studies according to PRISMA standards [10] is reported in Figure 1. Details of the 35 papers satisfying the inclusion criteria are summarized in Table 1.

### 2.2. Epidemiological Findings

The most common families of snakes involved in fatal SBE were *Viperidae* (*n* = 28–50.000%) followed soon after by *Elapidae* (*n* = 23–41.071%) and *Lamprophiidae* (*n* = 1–1.786%). In four cases (7.143%) no additional information about the snake was available. Most of the fatal cases of SBE reported can be classified into category 1 (*n* = 28) according to the WHO Guidelines on production, control, and regulation of snake antivenom immunoglobulins [8]. Just in two cases [22,33], the snake was not included in that list and in both cases, the snake was kept in captivity far from his habitat.

Most of the SBE victims were males (*n* = 35 cases; 62.500%) with a male/female ratio (M/F) of 1.67. Male victims were older than females. The median age in years for males was 40.77 and for females 28.71. The overlapping mean age was 36.25 years.

The body part most frequently bitten by snakes was the lower limb in 31 cases (55.357%), the upper limbs in 15 cases (26.786%), in one case the face (1.786%), and in one other case the genitalia (1.786%). Unfortunately, in eight cases (14.286%) the site of the snakebite was not reported. The vast majority of the SBE was accidental, but in one case study involving three victims, the manner of death was assessed as homicide [31].

The largest number of victims (*n* = 12) is reported from Australia, followed by Myanmar and India (*n* = 7 each), Brazil and Sri-Lanka (*n* = 6 each). However, all continents in the world are represented: 41.071% of victims were from Asia (*n* = 23), 21.429% from Oceania (*n* = 12), 19.643% from America (Northern, Central, and Southern; *n* = 11), 14.286% from Africa (*n* = 8), and 3.571% from Europe (*n* = 2). It is worth mentioning that 16 out of 56 cases were related to work activities. Most of the cases occurred in a rural context (countryside, paddies, etc.).

Regarding clinical history, the time interval between snakebites and death ranges from 25 min up to 35 days. Deaths occurred mostly after 24h for 22 cases (39.286%) and before 6h in 19 cases (33.929%). Most of the victims, 40 out of 56 cases (71.429%), were hospitalized and the specific antivenom was administered to 26 individuals. In a few cases, the victim was at first treated by a local healer or with traditional remedies [15,20,28,43].

### 2.3. Post Mortem Examination Findings

Most of the bite marks (19 out of 56 cases) are described as two puncture wounds from the animal’s fangs. Just a single puncture wound has been found in three cases, sometimes scratched or lacerated by the movement of the victim and/or his clothing deviating the fangs [44]. Additional wounds caused by other teeth or additional bites have been also described [29,31,43]. In two cases there were three fang marks [24,33]. Wankhede (2004) [44] described an unusual case in which four puncture wounds due to a single snakebite were found. In this strange case, the presence of the fang marks was consistent with a snakehead bigger in size than usual where the old fangs were still present, along with two new fangs.

In this regard, it is worth mentioning that for the identification of the offending snake the number of puncture wounds must be related to the number of teeth, which is unfortunately not the same in all kinds of snakes. Different species of snakes can have different anatomical conformation of the teeth and fangs. Fangs are sharp, long, hollow, or grooved teeth that are connected to a small sack full of venom in the snake’s head behind its eyes but not all snakes have fangs. Most snakes have six rows of teeth in total: one row on each lower jaw and two rows on each maxillary and palatine or pterygoid bones of the upper jaw [46]. It is also important to highlight that the victim’s clothes or gloves can represent an obstacle to the penetration of a fang [41].

With regard to the morphology of the fangs, sometimes the bite injuries are also described as linear or curvilinear stripes [15] fresh linear or multiple punctate abrasion [29]. The morphology of this kind of injury is quite similar to those produced by sharp force [47]. Snakebites in the multiple punctate abrasion pattern are often the result of the typical chewing activity of the coral snake. Instead, in most of the pit viper bites one or more fang marks are usually found [48] due to the stabbing motion of this snake specie’s bite [29]. Although important for the identification of the offending snake, only in very few cases (*n* = 8) [3,12,20,22,24,37,44,45], the distance between the fang marks is reported. At the external examination, the area surrounding the bite mark shows dermal alterations represented mostly by signs of flogosis such as edema, cellulitis, and erythema. Bruises related to the snake bites are also reported due to the bleeding into the skin. It is strongly recommended to check the signs of the vital reaction of the bite mark and to take samples from bitten tissue. Subcutaneous hemorrhages are pretty common at the puncture skin site along with skin necrosis caused by the snake venom components. Only very few cases report additional dermal effects of the venom such as multiple blisters [32], a rash [41], and a purpuric area due to microthrombosis of the skin [42]. The main histological findings are subcutaneous hemorrhage, rhabdomyolysis, and necrosis of the dermis. Autopsy findings are mostly non-specific general pathologic changes and consistent with a rapid death such as multi-visceral congestion or edema and petechial hemorrhages. In the brain, other than congestion and edema, petechial hemorrhages from pin-point size to large hematoma in the white matter have been reported in 13 cases out of 56 in total (23.214%) [14,15,16,18,20,23,24,25,27,39,44]. Microscopically, signs of ischemic and hemorrhagic encephalopathy have been found in six cases out of 56 [14,24,26,28,34,36] and extensive perivascular demyelination in a single case [21] Myocardial hemorrhages have been reported in seven cases out of 56 (12.500%) [12,14,16,27,35,39] and alterations consistent with myocardial infarction in a single case [14]. Signs of rapid failure of the left ventricle were mostly represented by pulmonary edema and congestion. Additional histological findings are hyaline membranes due to diffuse alveolar damage (DAD) in three cases [16,24,25] and widespread microthrombosis consistent with a disseminated intravascular coagulation (DIC) in four cases (7.142%) [12,19,20,39]. Renal injuries other than vascular congestion were mainly represented by tubular and/or glomerular necrosis found in 13 cases out of 56 in total (23.214%) [11,15,19,21,24,26,37,39,42]. Cortical and glomerular necrosis has been reported in one single case [30] and widespread ischemic necrosis in a different one [43]. Microthrombosis of the small inter-lobular vessels was found in a single case. Additional renal alterations are represented by acute interstitial nephritis (AIN) in three cases [3]. Intestinal injuries are reported in only three cases, but no better described [20,30,42]. Pituitary hemorrhage/necrosis and adrenal gland hemorrhage were present in another three cases [39].

## 3. Discussion

SBE is an acute medical emergency and a major public health problem that kills thousands of people each year, particularly in rural territories [49]. Most of the case studies considered in the present retrospective study were coming from tropical countries such as Brazil, Congo, India, Martinique, Myanmar, Nigeria, and Sri Lanka. The majority of fatal SBE involved snakes belonging to the Elapidae family (e.g., kraits—*Bungarus* spp., cobras—*Naja* spp., taipans—*Oxyuranus* spp., coral snakes—*Micrurus* spp., death adders—*Acanthophis* spp., and tiger snakes—*Notechis* spp.) and to the Viperidae family (e.g., rattlesnakes—*Crotalus* spp., Russell’s viper—*Daboia russelii*). According to the geographical latitudes, the species of snakes causing the largest numbers of bites and fatalities are *Echis* sp. (saw-scaled vipers) in Northern Africa, *Bothrops asper* and *B. atrox* (lance-headed pit vipers) in Central and South America, and *Naja* sp. (cobras) and *Bungarus* sp. (kraits) in Asia [50]. SBE is both a method of hunting and a means of protection [51]. Snake venom is produced by specialized venom glands located in the upper jaw and associated with fangs, which are specialized long teeth with a superficial groove, along which the venom runs. Sometimes the fangs can also have an enclosed canal, down which the venom flows inside the tooth [52]. The severity of SBE depends on several factors which include bite localization, snake’s size, condition of glands and teeth, bite angle and bite duration, microflora of the snake’s mouth and victim’s skin, age and weight of the victim, health status, victim’s activity after a bite [53,54,55].

However, the main deadly factor is represented by the components of the venom. Snake venoms are mixtures of protein families, and each of these families contains many different toxins or toxin isoforms [56]. A very recent proteomic study on the venom of the viperid genus indicates the presence of proteins belonging to at least 12 families, with a predominance of phospholipases A2 (PLA2s) (54%) and metalloproteinases (21.5%) [57]. In order to perform rapid detection and quantification of venom antigens, commonly found in higher concentrations from wound swabs or wound aspirates (within 15–30 min after bite) compared to body fluids, commercial highly sensitive venom detection kits are available [58] but they cannot distinguish between venoms of closely related species. Moreover, the detection of venom in a wound swab is not enough evidence that the patient has been envenomed, then also serum and urine samples should be stored for enzyme immunoassays [59,60,61,62], liquid chromatography–mass spectrometry with time-of-fight (LC–TOF/MS), DNA fingerprinting, and antibody microarrays [63]. Highly specific methods such as the detection of venom gland mRNA by reverse-transcription PCR [64,65] or snake-derived DNA in bite-wound swabs [66] are being developed.

Based on their effects, snake venoms can be classified as hemotoxic, neurotoxic, or cytotoxic and they can act all together involving multiple tissues and organs [51]. Neurotoxicity is a well-known effect of SBE by snakes of the Elapidae family (e.g., cobras, kraits, mambas, Australasian species, and sea snakes) but also of the Viperidae family (e.g., rattlesnakes, lance-headed pit vipers, and true vipers) [67]. Neurotoxic envenomation can cause a broad spectrum of early clinical manifestations varying from ptosis and ophthalmoplegia to paralysis of respiratory muscles, which causes asphyxia. Elapid venom can interfere with transmission across the motor end-plate as it can bind to the postsynaptic nicotinic receptors that would normally bind acetylcholine [14]. Delayed neurotoxic manifestations are represented by peripheral neuropathies, optic neuritis, and cortical blindness [68]. Cerebellar involvement, ataxia, and “locked-in syndrome” are very uncommon but they have been also described [68]. Outcomes are pretty good when prompt treatment has been provided.

Usually, snakes from the Viperidae family are considered hemotoxic [51]. Viperidae snakes were involved in most of the fatal SBE reviewed (28 cases out of 56 in total) followed soon after by Elapidae snakes (23 cases out of 56). Therefore, the 56 victims reviewed in this retrospective study suffered mostly hemotoxic and neurotoxic effects. Hemotoxic effects act on the heart and cardiovascular system and they can promote or inhibit hemostatic mechanisms including coagulation in different ways [53]. Hemotoxins can be pro-coagulant proteases such as prothrombin activators, thrombin-like enzymes, factor X, and factor V activators but also anticoagulant proteases like the factor IX and X inhibitors, protein C activator, anticoagulant PLA2s [69]. The venom components acting on fibrinolysis are fibrinolytic enzymes and plasminogen activators [69]. Bleeding disorders can occur everywhere in the body from the bite site to the internal organs. Major complications related to hemotoxic effects include life-threatening diseases like acute kidney injury (AKI), ARDS, or its less serious variant acute lung injury (ALI), capillary leak syndrome (CLS), and DIC [70]. Victims suffering from cytotoxic envenoming are characterized by painful and progressive swelling at the bite site, developing further into blistering and bruising, which is sometimes coupled with systemic effects including hypovolemic shock [51]. Tissue necrosis is one of the major problems in patients with SBE [71]. Anaphylactic shock secondary to SBE has been reported in very few cases [72,73,74]. In suspected anaphylactic or anaphylactoid episodes related to drugs or venoms, a careful and complete review of the clinical history and the results of allergy testing might be useful in making the diagnosis or distinguishing them from other causes [75].

In the cases included in our review, the gross findings were non-specific. Autopsy findings reported are mostly non-specific pathologic changes consistent with a rapid death such as multi-visceral congestion, edema, and petechial hemorrhages. Signs of ischemic and hemorrhagic encephalopathy can be found mostly represented by intracranial or subarachnoid hemorrhages as well as myocardial hemorrhages. Widespread microthrombosis has been also described as the result of the coagulation disorders caused by the hemotoxic effects of SBE [76]. A relationship between SBE and myocardial infarction (MI) can also occur [77]. In particular, after SBE different factors such as coronary thrombosis due to the procoagulant factors in venom, direct toxic effect on cardiomyocytes, decreased oxygen-carrying capacity of blood due to hemolysis, coronary vasoconstriction brought about by endothelins and safatoxins in venom, and myocardial hemorrhage and microvascular thrombin deposition can determine an acute myocardial injury due to an imbalance between myocardial oxygen supply and demand [77,78].

Other than pulmonary edema and congestion related to acute cardiac failure, pulmonary injuries by SBE are mostly represented by DAD and widespread microthrombosis in the DIC scenario. In particular, the effects of *Crotalus durissus terrificus* venom (CdtV) on the pulmonary mechanic events have been studied in male swiss mice [78,79]. The histological analysis has reported perivascular/interstitial pulmonary edema 6 h after venom injection and pulmonary hemorrhages 12 h after. The microscopic quantitative analysis has also reported a significant increase of pulmonary inflammation 3 and 6 h after venom injection, returning to control values 24 h after [79]. The systemic inflammatory response and the mechanisms of pulmonary injuries by *Crotalus durissus cascavella* venom have been also investigated in mice by Azavedo et al. (2020) [80]. They reported a significant increase of peribronchial inflammatory infiltrates, emphysema, and focal atelectasis indicative of severe pulmonary inflammation 3 h after venom injection followed by a decrease in the activity of inflammatory response, despite an increase in vascular congestion, alveolar septal thickening, and emphysematous areas at 6 h. Between 12 and 48 h, there were perceptible inflammatory infiltrates in the pulmonary parenchyma, hemorrhagic focuses, vascular congestion, and bronchial muscle distention [80].

Acute nephrotoxicity is a relevant complication of SBE mostly represented by AKI that is a major cause of morbidity and mortality. AKI following SBE is mostly related to ATN or ACN and has multifactorial pathogenesis including, hypotension, coagulopathy, intravascular hemolysis, thrombotic microangiopathy (TMA), and DIC, capillary leak syndrome, rhabdomyolysis, complement activation, and sepsis [81]. Once developed ATN, AKI, and death can follow quickly [81,82]. A total of 22 cases out of a series of 121 snake bite-induced AKI have been studied histologically [83], 91% of the study sample mostly after SBE by Viperidae family showed signs of ATN classified as severe in 55% of the cases. Tubulointerstitial lesions represented, in particular, by ATN, were observed in 70% to 80% of patients with AKI [83]. However, kidney injuries have been also found in three cases of SBE by Bungarus Fasciatus, Elapidae family [3]. Among the intestinal injuries, bowel ischemia due to thrombosis of the mesenteric vessels has been reported in very few cases. [84,85]. Pituitary hemorrhage/necrosis and adrenal gland hemorrhage are additional autopsy findings reported [55].

## 4. Conclusions

SBE is a public health issue in tropical countries. The identification of snake species can be crucial for appropriate clinical management and can improve the prognosis. Although snake venom can be easily detected by toxicological analysis, diagnosis of SBE related death is challenging for the forensic pathologist. The cause of death from SBE is primarily related to the neurotoxic, cytotoxic, and hemotoxic effects of the venom components including the paralysis of respiratory muscles, which causes asphyxia and hypoxic-ischemic encephalopathy, hemorrhagic shock, cardiomyopathy, ATN, and death from anaphylactic shock [15]. Key elements are still represented by investigation of the bite mark and circumstantial data. Although rare in the European context, [86,87,88] it is important for the forensic pathologist to be aware of the details of the bite experience, local and systemic symptoms, and to check the bite marks by taking samples from bitten tissue, mostly located in the lower limbs.

## 5. Materials and Methods

The review of the literature was conducted independently by two examiners and carried out according to the PRISMA statement [10].

### 5.1. Literature Search

This retrospective study was performed using the following electronic databases: PubMed, Scopus, and Web of Science. The following keywords were searched in all fields: “snake bite” AND “autopsy” OR “snakebite” AND “autopsy” to recognize relevant research available until 18 April 2020. No time limit was set. The systematic search was further extended by snowball search and hand searching by using Google Scholar.

### 5.2. Inclusion and Exclusion Criteria

The following inclusion criterion was adopted: (1) papers reporting autopsy cases of fatal SBE with a description of the autopsy and/or histological findings. Exclusion criteria comprised the following: (1) animal studies; (2) scientific article not published in English, and (3) full text not available. For duplicate studies, only the article with more detailed information was included.

### 5.3. Data Extraction

Two examiners independently provided the initial selection of the articles to determine whether they might potentially fit the inclusion criteria. The title, abstract, and full text of each potentially pertinent study were reviewed. Disagreements on the eligibility of the studies were solved between the two examiners; if no agreement could be reached, it was resolved using a third co-author as a reviewer. A data extraction sheet was developed and for each selected study the following items were recorded: snake species; details of the victims (sex; age); location (country, working place); clinical history (time interval between snakebite and death; admission to hospital; administration of antivenom); autopsy features (morphology of the bite; macroscopic and microscopic features of the bite site; macroscopic and microscopic findings at autopsy).

## Figures and Tables

**Table 1:** Summary of the items recorded from the 35 papers included in the retrospective study.

References	Country	Victim Age/Sex	Work-Related	Hospital Admission / Antivenom	Time Interval Bite-Death	Snake *Family*	Bite Area Site Macroscopical Findings	Bite Area Histological Findings	Autopsy Macroscopical Findings	Autopsy Histological Pattern
Barraviera B et al., 1989 [11]	Brazil	60/M	n.a.	Yes/Yes	4–5 d	Rattlesnake *Viperidae*	Right leg. Ulceration covered with a blood-stained scab.	Local edema and inflammatory signs. Focal myonecrosis.	n.a.	K: acute tubular necrosis
Barraviera B et al.,1989 [11]	Brazil	59/M	n.a.	Yes/Yes	5 d	Rattlesnake *Viperidae*	Right leg. Two punctiform erythematous lesions.	No signs of inflammation.	n.a.	Li: parenchymal necrosis.
Benvenuti LA et al., 2003 [12]	Brazil	36/F	n.a.	Yes; No	45 min	Bothrops jararacussu *Viperidae*	Left ankle. Two puncture wounds 25 mm apart. Ecchymosis on the left leg along the great saphenous vein.	Local hemorrhage, thrombosis, and coagulative necrosis of the subcutaneous and muscular tissue.	L: hemorrhage.	PG: normal. H: fibrosis and interstitial hemorrhage. L: extensive alveolar hemorrhage and edema with numerous platelet thrombi in capillaries and venules. DIC.
Blaylock RSM et al., 2002 [13]	South Africa	40/M	Yes	Yes; Yes	1 h 15 min	Puff Adder (Bitis Arientas) *Viperidae*	Left wrist. Single puncture wound.	Extensive hemorrhage between muscle bundles and connective tissue.	H: myocardium soft and flabby with hemorrhagic RA. L: edema and congestion. Li; K: pale aspect.	n.a.
Blumenthal R et al., 2019 [14]	South Africa	27/M	Yes	Yes; Yes	3 d	Black Mamba (Dendroaspis polylepis) *Elapidae*	Right hand. Single puncture mark.	Subcutaneous hemorrhage. Minimal skin necrosis and hemorrhage at the puncture site.	Diffuse fresh hemorrhages around the vocal cords, the epiglottis, and in the LV. H: petechiae overlying the anterior and posterior epicardial.	B: hypoxic-ischemic encephalopathy. H: diffuse hemorrhages. L: fibrinosuppurative consolidation.
Chen L et al., 2013 [15]	China	43/M	n.a.	Yes (at the end); n.a.	5 d	Agkistrodom halys*Viperidae*	Right foot. 0.3 cm-long laceration made of two pairs of thin stripes. Right foot swollen and ecchymotic.	Subcutaneous hemorrhage and cellulitis.	Diffuse edema and congestion.	K: Toxic nephropathy, proximal tubular epithelial degeneration, and necrosis, protein and pigment cast in the distal tubules.
Chen L et al., 2013 [15]	China	4/M	n.a.	Yes; n.a.	2 d	Agkistrodom halys*Viperidae*	Right foot. 1.2 × 0.5 cm snakebite wound.	n.a.	Diffuse edema and congestion.S: anemic aspect.	B, T, K: congestion. H, L: edema and congestion.
Curry SC et al., 1985 [16]	USA	44/F	No	Yes; Yes	15 d	Rattlesnake *Viperidae*	Right calf. Two fang marks surrounded by an area of discoloration.	Area of tissue disruption leading toward a vein. Small eschar over the site of fang penetration through epidermidis.	B, Li, S: enlarged and congested. H: lightly dilated and flabby; superficial hemorrhages. L: expanded and indurated.	B: focal hemorrhages in the leptomeninges. L: severe ARDS. K: eosinophilic proteinaceous casts in small tubules.
Karlawad M et al., 2017 [17]	India	75/M	Yes	Yes; Yes	3 h	n.a.	Scrotum. Two punctured wounds surrounded by an area of discoloration.	Ulcerated stratified squamous epithelium with sub-epithelial tissue showing congested blood vessels and edema.	Diffuse congestion.	L: interstitial and intra-alveolar edema with rupture of alveoli. H: unremarkable.
Keith J et al., 2012 [18]	Australia	69/F	No	Yes; Yes	2 d	Australian Brown Snake (Pseudonaja textilis) *Elapidae*	Foot.	n.a.	B: swollen with tonsillar herniation and V-shaped hemorrhage within the cerebellum.	Unremarkable.
Kitchens CS et al., 1987 [19]	USA	67/M	No	Yes; Yes	2 d 12 h	Canebrake Rattlesnake (Crotalus Horridus Atricaudatus) *Viperidae*	Left Hand. Two puncture wounds.	Rhabdomyolysis. The left thenar muscle disclosed a sparse infiltration of PMNs.	M: soft consistency underlying the bite.	H: patchy myocardial necrosis. L: hemorrhagic pulmonary edema; small scattered pulmonary thrombi consistent with terminal DIC. K: massive acute tubular necrosis. M: rhabdomyolysis of the skeletal muscles.
Malbranque S et al., 2008 [20]	Martinique	74/M	Yes	Yes; Yes	10 d	Fer-de-Lance (Bothrops lanceolatus) *Viperidae*	Left elbow. Fang puncture marks 20 mm apart surrounded by a swollen area.	n.a.	Serous effusions in the peritoneal, pericardial, and pleural cavities. B: edema with petechiae. H: infarcts of different ages and sizes. L: edematous and congested.	B: ischemic changes; thrombotic lesions were confined to the sub-arachnoid space. H: Fibrinous pericarditis and scattered ischemic foci in the LV. L: thrombotic lesion within the small pulmonary arteries and the capillaries of the inter-alveolar septa. K: thrombotic lesion within the small renal inter-lobular vessels and, to a localized extent. I: thrombotic lesion in the small intestine and colon.
Malhotra P et al., 2005 [21]	India	27/F	n.a.	Yes; Yes	n.a.	n.a.	Left foot. Fang marks.	n.a.	Unremarkable.	B: extensive perivascular demyelination and limphocyte cuffing. K: acute tubular necrosis.
Marsh N, et al., 2007 [22]	USA (captivity)	33/F	No	No; No	n.a.	Gaboon viper (Bitis gabonica) *Viperidae*	Left hand. Two puncture wounds 11–12 mm apart surrounded by a swollen and bloody area.	n.a.	Ecchymoses of the head, trunk and extremities, the periorbital areas, and left orolaryngeal/mandibular angle. L: hemorrhagic appearance and congestion.	n.a.
McGarity BH et al., 1991 [23]	Australia	40/M	n.a.	Yes; Yes	2 d	Tiger snake (Notechis scutatus) *Elapidae*	Left heel.	n.a.	B: edematous; right tentorial and subfalcial herniation; massive right intracerebral hemorrhage, with rupture into the right basal ganglia and cerebellum.	n.a.
Milani R Jr et al., 1997 [24]	Brazil	3/F	No	Yes; Yes	18 h 40 min	Jararacuçu (Bothrops jararacussu) *Viperidae*	Left thigh. Three widely spaced fang puncture marks.	Extensive rhabdomyolysis with hemorrhagic foci. Hemorrhage and necrosis in the dermis and hemorrhage in subcutaneous fat near the site.	B: edema.	L: hemorrhages with inflammatory infiltration of neutrophils in septa and alveoli and deposition of intra-alveolar fibrin. Li: hepatic sinusoids and portal tracts infiltrated with PMNs. P: fatty necrosis with hemorrhage of the parenchymal cells.
Milani R Jr et al., 1997 [24]	Brazil	65/M	n.a.	Yes; Yes	4 d 9 h	Jararacuçu (Bothrops jararacussu) *Viperidae*	Left calf. Two fang punctures surrounded by a ring of bruising.	Necrosis of muscle fibers and interstitial hemorrhage in the region of the wound.	B: subarachnoid petechiae and edema.	B: ischemic necrosis of the mucosa and in small cortical and meningeal vessels with local meningeal hemorrhage and cerebral edema. Li: fatty (alcoholic) liver with evidence of shock. I: fibrin thrombi in small sub-mucosal vessels. AG: focal hemorrhages in the medulla, with depletion of the zona fasciculata. K: acute tubular necrosis DIC.
Moar JJ et al., 2016 [25]	South Africa	23/M	Yes	Yes; No	Several hours	Rinkhal (Hemachatus haemachatus) *Elapidae*	Right forearm. Two bite marks each 3 mm in diameter and 1.5 cm apart.	Displaced and disrupted fragments of adnexal structures into the depths of the wound, together with hemorrhaging into the surrounding interstitium, and compression of the collagen bundles of the dermis with collagen displacement and disruption.	B, L: edema and petechial hemorrhages.	B: Extravasation of erythrocytes into the surrounding Virchow–Robin spaces. H: in LV marked interstitial vascular dilatation and congestion with focal subendocardial papillary muscle fibrosis. L: ARDS. Li: portal vessels congestion and widening by reticulin and a portal triaditis. S: sinusoidal dilatation and congestion; mantle zone hyperplasia. K: interstitial vascular and glomerular capillary loop dilatation and congestion with a chronic inflammatory cell infiltrate.
Namal Rathnayaka RMMK et al., 2018 [26]	Sri Lanka	42/M	Yes	Yes; No	16 d	Hump nose Viper (Hypnale Hypnale) *Viperidae*	Right foot.	n.a.	B: a small infarct (1.5 cm–1 cm) in the basal ganglia. H: reduced lumen of the left coronary artery.	L: hemorrhages. K: acute tubular necrosis. H: normal.
Namal Rathnayaka RMMK et al., 2019 [27]	Sri Lanka	66/M	No	Yes; No	4 d	Hump nose Viper (Hypnale Hypnale) *Viperidae*	Left foot. Two fang punctures.	n.a.	B, H, L: hemorrhages.	L: edema and hemorrhage.
Namal Rathnayaka RMMK et al., 2017 [28]	Sri Lanka	43/M	No	Yes; Yes	11 d	Russell’s viper (Daboia russelii) *Viperidae*	Right foot. Swelling of the leg.	n.a.	B: infarcted area of parietal lobes. K: petechial hemorrhages.	B: hemorrhages, thrombi, and congested blood vessels. H, L, K: normal.
Norris RL et al., 2009 [29]	USA	38/M	No	No; No	2 h	Coral snake (Micrurus fulvius) *Elapidae*	Right Hand; Left Arm. Fresh 6 mm linear abrasion and multiple punctuate abrasions in a small group.	n.a.	L: congestion.	Unremarkable.
Okamoto O et al., 2017 [30]	Japan	84/F	Yes	Yes; Yes	35 d	Mamushi (Gloydius Blomhoffii) *Viperidae*	Right elbow.	n.a.	Li: acute necrosis. I: extensive patchy necrosis at the small intestine; multiple ulcers and bleeding at the ascending colon. K: cortical necrosis.	Li: necrosis around the central vein. I: necrosis extended to the muscle layer. K: glomerular necrosis.
Paulis MG et al., 2016 [31]	Egypt	9/F	No	No; Yes	n.a.	Egyptian Cobra (Naja Haje) *Elapidae*	Right leg. Five pairs of fang marks with almost fixed distance between the paired ones, all surrounded by erythema and little swelling.	n.a.	Internal organ congestion.	n.a.
Paulis MG et al., 2016 [31]	Egypt	6/F	No	No; No.	n.a.	Egyptian Cobra (Naja Haje) *Elapidae*	Left leg. Two pairs of fang marks, each pair was symmetrical, all surrounded by erythema and little swelling.	n.a.	Internal organ congestion.	n.a.
Paulis MG et al., 2016 [31]	Egypt	4/F	No	No; No.	n.a.	Egyptian Cobra (Naja Haje) *Elapidae*	Right leg. Multiple fang marks close to each other surrounded by erythema.	n.a.	Internal organ congestion.	n.a.
Pramod Kumar GN et al., 2013 [32]	India	68/M	n.a.	Yes; n.a.	4 d	n.a.	Right thigh. Bluish black discoloration of the outer aspect of the right thigh and of the right leg. Multiple blisters and ulcerative necrosis. Extravasation of blood in the subcutaneous plane.	n.a.	Unremarkable (coexistence of gastric and kidney cancers).	n.a.
Quain, 1852 [33]	England (captivity)	30/M	Yes	Yes: No.	1 h 30 min	Hooded Snake - Cobra de Capello (Naja tripudians) *Elapidae*	Face. Three puncture marks surrounded by an ecchymotic area.	n.a.	Congestion of all internal organs, particularly of the spleen.	n.a.
Rathnayaka RMMKN et al., 2017 [34]	Sri Lanka	53/M	n.a.	Yes; Yes	26 d	Russell’s viper (Daboia russelii) *Viperidae*	Right Leg. Swollen area.	n.a.	B: infarcted area of parietal lobes of the brain. K: reduced cortico-medullary demarcation in both kidneys and petechial hemorrhages.	n.a.
Sarkar N et al., 2018 [3]	India	27/M	No	No; No.	almost 6 h	Banded Krait (Bungarus faciatus) *Elapidae*	Hand. Two pin-point punctured wounds, subcutaneous deep, placed 8 mm apart.	n.a.	Internal organ congestion K: bilateral cortico-medullary hemorrhage and congestion.	K: interstitial hemorrhage; inflammatory cell infiltration and swelling.
Sarkar N et al., 2018 [3]	India	26/F	No	No; No	almost 6 h	Banded Krait (Bungarus faciatus) *Elapidae*	Right shoulder. Two pin-point punctured wounds.	n.a.	Internal organ congestion K: bilateral cortico-medullary hemorrhage and congestion.	K: interstitial hemorrhage and inflammatory cell infiltration.
Sarkar N et al., 2018 [3]	India	4/F	No	No; No	almost 6 h	Banded Krait (Bungarus faciatus) *Elapidae*	Right foot.	n.a.	Internal organ congestion. K: bilateral cortico-medullary hemorrhage and congestion.	K: increased cytoplasmatic eosinophilia and occasional surface blebbing of the renal tubular epithelial cell (early ischemic injury).
Silva A et al., 2013 [35]	Sri Lanka	19/M	No	Yes; Yes	1 d 22 h	Indian Krait (Bungarus caeruleus) *Elapidae*	n.a.	n.a.	H, L, K: Petechial hemorrhages and edema.	n.a.
Silva de Oliveira S et al., 2017 [36]	Brazil	59/F	No	Yes; Yes	2 d	Jaracaca do Norte (Bothrops atrox) *Viperidae*	Right foot. Ecchymotic and swollen area.	n.a.	B: Subarachnoid and intraparenchymal hemorrhage.	n.a.
Soe S et al.,1993 [37]	Myanmar	13/M	n.a.	n.a.; n.a.	n.a.	Russell’s Viper (Vipera Russelli) *Viperidae*	n.a.	n.a.	n.a.	K: Glomerular changes: endothelial swelling, increased cells; degeneration and necrosis of the tubules, and fibrin deposition.
Soe S et al.,1993 [37]	Myanmar	15/F	n.a.	n.a.; n.a.	n.a.	Russell’s Viper (Vipera Russelli) *Viperidae*	n.a.	n.a.	n.a.	K: Glomerular changes: endothelial swelling, increased cells; degeneration and necrosis of the tubules, and fibrin deposition.
Soe S et al.,1993 [37]	Myanmar	15/F	n.a.	n.a.; n.a.	n.a.	Russell’s Viper (Vipera Russelli) *Viperidae*	n.a.	n.a.	n.a.	K: Glomerular changes: endothelial swelling, increased cells; degeneration and necrosis of the tubules, and fibrin deposition.
Soe S et al.,1993 [37]	Myanmar	49/M	n.a.	n.a.; n.a.	n.a.	Russell’s Viper (Vipera Russelli) *Viperidae*	n.a.	n.a.	n.a.	K: Glomerular changes: endothelial swelling, increased cells; degeneration and necrosis of the tubules, and fibrin deposition
Sutherland SK, 1992 [38]	Australia	16/F	No	Yes; n.a.	1 h	Brown snake (Pseudonaja Textilis) *Elapidae*	Right heel. Fang marks 5 mm apart.	n.a.	Unremarkable.	n.a.
Sutherland SK, 1992 [38]	Australia	27/F	n.a.	Yes; n.a.	1 h 30 min	Brown snake *Elapidae*	n.a.	n.a.	Unremarkable.	n.a.
Sutherland SK, 1992 [38]	Australia	61/F	NO	Yes; No	1 h 30 min	Brown snake *Elapidae*	Right ankle.	n.a.	Small retroperitoneal hemorrhage.	n.a.
Sutherland SK, 1992 [38]	Australia	51/M	n.a.	Yes; Yes	1 h	Taipan *Elapidae*	n.a.	n.a.	Unremarkable.	n.a.
Sutherland SK, 1992 [38]	Australia	2/F	No	No; No	n.a.	Brown snake *Elapidae*	Left leg. Two minute puncture wounds.	n.a.	L: congestion and edema with numerous petechial hemorrhages on the visceral pleurae.	n.a.
Sutherland SK, 1992 [38]	Australia	42/M	No	Yes; Yes	5 h 30 min	Taipan *Elapidae*	n.a.	n.a.	Unremarkable.	n.a.
Sutherland SK, 1992 [38]	Australia	60/M	Yes	Yes; Yes	25 min	Brown snake *Elapidae*	Right Foot.	n.a.	Unremarkable.	n.a.
Sutherland SK, 1992 [38]	Australia	35/M	No	No; No	35 min	Brown snake (Pseudonaja Textilis) *Elapidae*	Right hand.	n.a.	H: Coronary arteries disease. Li: cirrhosis.	n.a.
Sutherland SK, 1992 [38]	Australia	48/F	No	No; No	30 min	n.a.	Right Foot. Two tiny puncture wounds.	n.a.	n.a.	Quantities of venom in urine that reacted to tiger snake venom.
Than-Than et al., 1989 [39]	Myanmar	19/M	Yes	Yes (at the end); Yes	14 h	Russell’s Viper (Vipera Russelli Siamensis) *Viperidae*	Ankle. bruising area.	n.a.	L: petechial hemorrhages.	PG: marked acute congestion. L: intraseptal capillaries were congested and contained fibrin thrombi as did some of the large pulmonary vessels, erythrocytes in the alveoli but no definite hemorrhage. S: red pulp congested with pseudoamyloid change in the central arterioles of some of the malpighian corpuscles. Li: congested with some nonspecific inflammatory cells. K: the glomerular tufts were congested and some contained fibrin thrombi; early tubular necrosis. B, H, AG: normal.
Than-Than et al., 1989 [39]	Myanmar	17/M	Yes	Yes (at the end); Yes	1 d 12 h	Russell’s Viper (Vipera Russelli Siamensis) *Viperidae*	Foot.	Hemorrhage, necrosis, and the presence of fibrin thrombi in small vessels. Subcutaneous fibrofatty tissue heavily infiltrated by a mixture of PMNs, leucocytes, lymphocytes, and eosinophils. Dense acidophilic homogeneous fibrin-like material in the surrounding blood vessels.	Serous fluid in the pleural and peritoneal cavities. B: edema. PG, AG: diffusely hemorrhagic. H, L: petechial hemorrhages. K: demarcation between dark red medulla and pale cortex.	PG: congestion and hemorrhage with foci of fibrinous material. H: numerous focal hemorrhages on the epicardial and endocardial surfaces. L: intense capillary wall congestion. AG: patchy congestion and focal hemorrhages. K: glomerular congestion; marked congestion of the medulla and corticomedullary junction with interstitial hemorrhages; early tubular necrosis.
Than-Than et al., 1989 [39]	Myanmar	15/F	Yes	Yes; Yes	2 d 4 h	Russell’s Viper (Vipera Russelli Siamensis) *Viperidae*	Foot.	n.a.	B: edematous and congested. Large blood clot (5 × 12 mm) in the pituitary fossa. PG: dark grey in color and hemorrhagic parenchyma. L, H, St: subserosal hemorrhage. AG: hemorrhagic. K: Blood clots in the pelvises. Contrasting red medulla and pale cortex.	B: edematous with dilatation of perineuronal and Virchow-Robin spaces. PG: extensive areas of hemorrhage and intense congestion of the sinusoids. L: acute alveolar wall congestion without hemorrhage, fibrin thrombi or edema. S: expansion of the red pulp. K: glomerular congestion with slight increase in cellularity. Early tubular necrosis.
Tibballs J et al., 1991 [40]	Australia	11/M	No	Yes; Yes	Less than 1 d	Tiger snake (Notechis scutatus) *Elapidae*	Left wrist.	n.a.	B: multiple intracerebral hemorrhages and subarachnoid collections of blood.	n.a.
Tilbury CR et al., 2016 [41]	Congo	46/M	Yes	Yes; Yes	2 h 20 min	Burrowing Asps (Atractaspis corpulenta) *Lamprophiidae*	Left hand. Punctuation-shaped wound (!), 2.5 mm in length surrounded by a non-specified rash.	Disruption of the epidermal and dermal tissue layers, with associated fresh hemorrhage.	Diffuse congestion of the organs. H: flabby. L: edematous and congested.	H: acute ischemic changes, foci of contraction band necrosis with no accompanying inflammatory response. L: prominent pulmonary edema and severe pulmonary congestion.
Tranca S et al., 2016 [42]	Romania	56/M	n.a.	Yes; Yes	n.a.	Vipera Berus *Viperidae*	Right tight. Two puncture wounds surrounded by a purpuric area.	Diffuse epidermal necrosis associated with micro-hemorrhages and micro-thrombosis in the dermis and hypodermis.	H, L: edema.	B: edema and hemorrhagic lesions in the leptomeninges. H: myocardial fibrosis, epicardial and interstitial hemorrhages. L: atelectasia and pulmonary hemorrhagic edema. Li: diffuse hepatic necrosis. I: enteral necrosis and acute mucosal hemorrhages. K: acute tubular necrosis.
Varagunam T et al., 1970 [43]	Sri Lanka	30/M	Yes	Yes; No	11 d	Pit-Viper (Agkistrodom hypnale) *Viperidae*	Left hand. Scars of two fang marks.	n.a.	K: larger, external surface was smooth with many areas of necrosis or hemorrhage; necrotic areas confined to the cortices; the medullae were congested. H, L, Li, S, I, AG: normal.	Li: early fatty changes. K: irregular areas of coagulative necrosis in the cortices; both glomeruli and tubules were involved in the necrotic process; Necrosis in many intralobular arteries and arterioles.
Wankhede AG, 2004 [44]	India	25/M	Yes	Yes, n.a.	1 d	Russell’s Viper (Vipera Russelli Siamensis) *Viperidae*	Right ankle. Two puncture wounds of diameter 1.5 mm each and 2.3 cm apart and puncture lacerated wounds one 7 mm below the lateral puncture wound, 1.5 cm in length, and the second 1.6 cm below the medial puncture wound 6 mm in length.	n.a.	B: petechial hemorrhages, congestion and edema. L: congestion and edema.	Unremarkable.
Warrell DA et al., 1976 [45]	Nigeria	10/M	No	No; No	2 h	Egyptian Cobra (Naja Haje) *Elapidae*	Right hand. Two fang punctures 2 cm apart, surrounded by a swollen area.	n.a.	Unremarkable.	B, H: normal.

AG: adrenal glands; ARDS: acute respiratory distress syndrome; B: brain; DIC: disseminated intravascular coagulation; H: heart; I: intestine; K: kidneys; L: lungs; Li: Liver; LV: left ventricle; M: muscle; n.a.: not available; P: pancreas; PG: pituitary gland; PMNs: polymorphonuclear leukocytes; RA: right atrium; S: spleen; St: stomach; T: thymus.
